# Class-Specific Evolution and Transcriptional Differentiation of *14-3-3* Family Members in Mesohexaploid *Brassica rapa*

**DOI:** 10.3389/fpls.2016.00012

**Published:** 2016-01-26

**Authors:** Ruby Chandna, Rehna Augustine, Praveena Kanchupati, Roshan Kumar, Pawan Kumar, Gulab C. Arya, Naveen C. Bisht

**Affiliations:** National Institute of Plant Genome ResearchNew Delhi, India

**Keywords:** 14-3-3, *Brassica rapa*, expression differentiation, gene divergence, polyploidy

## Abstract

14-3-3s are highly conserved, multigene family proteins that have been implicated in modulating various biological processes. The presence of inherent polyploidy and genome complexity has limited the identification and characterization of 14-3-3 proteins from globally important *Brassica* crops. Through data mining of *Brassica rapa*, the model *Brassica* genome, we identified 21 members encoding 14-3-3 proteins namely, BraA.GRF14.a to BraA.GRF14.u. Phylogenetic analysis indicated that *B. rapa* contains both ε (epsilon) and non-ε 14-3-3 isoforms, having distinct intron-exon structural organization patterns. The non-ε isoforms showed lower divergence rate (*Ks* < 0.45) compared to ε protein isoforms (*Ks* > 0.48), suggesting class-specific divergence pattern. Synteny analysis revealed that mesohexaploid *B. rapa* genome has retained 1–5 orthologs of each *Arabidopsis 14-3-3* gene, interspersed across its three fragmented sub-genomes. qRT-PCR analysis showed that 14 of the 21 *BraA.GRF14* were expressed, wherein a higher abundance of non-ε transcripts was observed compared to the ε genes, indicating class-specific transcriptional bias. The *BraA.GRF14* genes showed distinct expression pattern during plant developmental stages and in response to abiotic stress, phytohormone treatments, and nutrient deprivation conditions. Together, the distinct expression pattern and differential regulation of *BraA.GRF14* genes indicated the occurrence of functional divergence of *B. rapa* 14-3-3 proteins during plant development and stress responses.

## Introduction

14-3-3 proteins derived their unique name from the studies of fractionation of bovine brain proteins on DEAE cellulose and their electrophoretic mobility on starch gel electrophoresis (Moore and Perez, 1967). These regulatory proteins are present in all eukaryotes and involved in protein interactions mediated signal transduction pathways. In plants, 14-3-3 proteins function by binding to numerous “client” proteins in a phosphorylation-dependent manner to modulate their activities, degradation, or sub-cellular localization (Rosenquist et al., 2001; Paul et al., 2012). So far, more than 300 putative 14-3-3 interacting client proteins have been reported in plants out of which nitrate reductase, sucrose-phosphate synthase, plasma membrane H^+^-ATPase, EmBP1, and VP1 transcription factors, the RSG transcription activator, a lipoxygenase from barley, a membrane bound ascorbate peroxidase and a outward-rectifying K^+^ channel in tomato are well-characterized (reviewed in Oecking and Jaspert, 2009; Denison et al., 2011; de Boer et al., 2013). This high number reflects the potential role of the 14-3-3s in controlling various signaling and developmental processes in plants. Current literatures clearly suggest the involvement of 14-3-3 proteins in various physiological processes including primary carbon and nitrogen metabolism (Comparot et al., 2003), abiotic and biotic stress responses (Roberts et al., 2002; Umezawa et al., 2004; Yan et al., 2004; Chen et al., 2006; Yang et al., 2013; Catalá et al., 2014; Zhou et al., 2014; He et al., 2015; Li et al., 2015), signaling pathways of phytohormones like ABA, GA, and BR (Testerink et al., 1999; Igarashi et al., 2001; Ryu et al., 2007; Kim et al., 2009; Zhou et al., 2015), and also during plant growth and development (Radwan et al., 2012; de Boer et al., 2013; Sun et al., 2014; van Kleeff et al., 2014).

To carry such diverse roles, almost all eukaryotes harbor multiple isoforms of *14-3-3* genes, with two present in yeast, seven in humans, and more than a dozen in vascular plants. The model monocot (*Oryza sativa*) and dicot (*Arabidopsis thaliana*) plant genomes encode 8 and 13 expressed *14-3-3* genes, respectively (DeLille et al., 2001; Rosenquist et al., 2001; Chen et al., 2006; Yao et al., 2007). Data mining of sequenced plant genomes has led to the identification of much higher number of *14-3-3* genes, particularly from polyploid genomes. A total of thirty-one *14-3-3* cDNAs encoding 25 unique proteins were identified from allotetraploid cotton (Sun et al., 2011), whereas the diploidized tetraploid soybean genome has eighteen *14-3-3* gene homologs, of which 16 are expressed (Li and Dhaubhadel, 2011). The chromosomal/segmental duplications and the evolutionary diversification are largely known to shape the quantitative variability of functional 14-3-3 proteins across plant species. Even though high sequence similarity exists among multiple copies of 14-3-3s, the specificity of these protein isoforms harboring definite subcellular localization, tissue-specific expression, and dynamic regulation in response to environmental changes is well-reviewed (Kjarland et al., 2006). Also each isoform, expressing in different subcellular location inside the cell, interacts with different client partners and relay the downstream signaling. This partly explains the versatility of so many isoforms in a plant species regulating a wide range of biological processes and functions.

*Brassica* species, the closest crop relatives to *Arabidopsis*, play an important role in global agriculture and horticulture. *Brassica rapa* (field mustard) is one of the globally important *Brassica* crop because of its enormous genetic and morphological diversity, and being utilized as leafy vegetables, vegetable oils, turnips roots, turnip greens, turnip tops, and fodder turnip. Besides, it is one of the diploid progenitor species (*n* = 10) which contributed the “A” genome to the important oilseed crops, *Brassica juncea* (*n* = 18, AABB) and *Brassica napus* (*n* = 19, AACC). Because of its pivotal position among the *Brassica* species, the recent sequencing of *B. rapa* genome offers an excellent opportunity to study the structural and functional evolution of candidate genes (The *B. rapa* Genome Sequencing Project Consortium; Wang et al., 2011). Sequence level studies although reflect high similarity in functional genes present between *Arabidopsis* and *B. rapa*, quantitative variation and evolutionary divergence in members of gene families present in polyploid *Brassica* genome, however, may contribute to the remarkable phenotypic plasticity and environmental adaptability of economically important *Brassica* species.

To investigate the important roles played by 14-3-3 protein isoforms in *Brassica* crops, comprehensive analysis of *14-3-3* gene homologs was undertaken from *B. rapa*. Present study through data mining of the recently sequenced *B. rapa* genome identified 21 divergent *14-3-3* genes providing their chromosomal and sub-genomic localization, phylogenetic relationship, and divergence analysis. We further carried out comprehensive expression profiling of 14 expressed *14-3-3* gene family members in *B. rapa* across plant development stages, abiotic stress conditions, phytohormone treatments and under nutrient deprivation conditions. We observed highly coordinated tissue- and condition-specific expression of *B. rapa 14-3-3* transcripts, suggesting their multifarious roles across plant growth, development, and environmental cues. The study provides an excellent base for conducting further in-depth research on various signaling pathways regulated by *B. rapa* 14-3-3 proteins, which could be utilized for agricultural improvements of the mustard crop.

## Materials and methods

### Plant materials and growth condition

*B. rapa* genotype YID1 was grown in a controlled growth conditions set as day (22°C, 10 h)/night (15°C, 14 h) cycle and 70% relative humidity. Different developmental stages namely, seedling, root, stem, leaf, and silique (20 days post anthesis), were harvested and immediately frozen in liquid nitrogen and stored at −80°C.

### Identification of the 14-3-3 isoforms and phylogenetic comparisons

*Arabidopsis 14-3-3* gene sequences were used to search against the *B. rapa* genome database (http://brassicadb.org/brad/; Cheng et al., 2011). The retrieved sequences were then named according to the existing nomenclature as *BraA.GRF14.a* to *BraA.GRF14.u*. Multiple sequence alignments of the encoded proteins were done using Clustal W and the phylogenetic trees were constructed using the Neighborhood-joining method of MEGA5 (Tamura et al., 2011). The human 14-3-3 theta isoform (NP_006817) was used as an out-group protein.

### RNA isolation and cDNA synthesis

RNA was extracted from plant tissues using the Spectrum Plant Total RNA Kit (Sigma Life Sciences, USA) according to manufacturer's instructions. The quantity and quality of RNA sample was checked using Nano spectrophotometer (ND-1000 Thermo scientific); and RNA samples with 260/280 ratio (1.9–2.1) and 260/230 ratio (2.0–2.5) were used for further analysis. First strand cDNA was synthesized by reverse transcribing 2 μg of total RNA with random primers of high-capacity cDNA Reverse Transcription kit (Applied Biosystems, USA) in a 20 μl reaction according to manufacturer's instructions. Diluted cDNA (1:50) was used for the real-time qRT-PCR reaction.

### Expression profiling of *B. rapa 14-3-3* genes

Real-Time PCR was performed using standard cycling conditions (95°C for 10 min, 40 cycles of 15 s at 95 and 60°C for 60 s) in final volume of 20 μl in a 7900 HT real time PCR machine (Applied Biosystems). Reaction mixture contained SYBR Green Master Mix (Kapa Biosystems), 10 pmol of gene-specific forward and reverse primers, and 2 μl of the diluted cDNA (~200 pg). To check for the specificity of PCR amplification dissociation curve was generated (Figure S3). The *Ct*-values were determined for each reaction using SDS version 2.3 and RQ manager version 1.2 (Applied Biosciences) software with default parameters. *GAPDH* and *ACT2* genes of *Brassica* origin were used as endogenous control (Chandna et al., 2012). Three independent sets of experiments were conducted with two technical replicates each to confirm results. Primers used for qRT-PCR analysis are tabulated in Table S4.

### Elicitor and stress treatments

For elicitor and stress treatments, seedlings were grown initially for 5 days on agar plates containing one-half strength Murashige and Skoog (MS) medium. Before elicitor induction, seedlings were adapted to liquid MS culture containing 1% sucrose for 24 h. For hormones induction, methyl jasmonate (MeJA, 0.2 mM), salicylic acid (SA, 0.2 mM), indole-3-acetic acid (IAA, 0.1 mM), and abscisic acid (ABA, 0.1 mM) were independently added to the medium (Chandna et al., 2012). Seedlings were also subjected to different stress conditions such as heat shock (37°C), cold (4°C), salinity (300 mM NaCl), and dehydration (between folds of tissue paper). The plants were harvested at 15 min, 30 min, 3 h, and 6 h durations and the mock treated seedling for same time interval served as control. For elicitor treatment experiments, the expression cut-off of 1.5-fold change (w.r.t. corresponding control) was used to identify the up- and down-regulated transcripts.

### Nutrient deprivation experiments

Plants were initially grown in MS agar medium for 3 days at 22°C with 10 h daylight at 200 μmol m^−2^ s^−1^. Seedlings were then transferred and adapted for 2 days on hydroponic nutrient solution containing 5 mM KNO_3_, 2.5 mM KH_2_PO_4_, 2.5 mM Fe-EDTA, 2 mM Ca(NO_3_)_2_, 2 mM MgSO_4_, and the following micronutrients: 1 mM NaCl, 1.4 mM MgCl_2_, 0.001 mM CaCl_2_, 7 mM H_3_BO_3_, 0.1 mM ZnSO_4_, 0.05 mM CuSO_4_, and 0.002 mM Na_2_MoO_4_. For nutrient deprivation experiments, the 2 days old hydroponically adapted seedlings were then transferred to nutrient solution lacking either Phosphorus (P), or Potassium (K), or Nitrogen (N). For the “P” deprivation, 2.5 mM KH_2_PO_4_ was replaced with 2.5 mM K_2_SO_4_. For the “K” deprivation, 5 mM KNO_3_ was replaced with 3 mM Ca(NO_3_)_2_; whereas 2.5 mM KH_2_PO_4_ was replaced with 0.25 mM K_2_SO_4_. For the “N” deprivation, 5 mM KNO_3_, and 2 mM Ca(NO_3_)_2_ were replaced with 5 mM KCl and 2 mM CaCl_2_, respectively. The untreated control and nutrient deprived seedlings were harvested at 1, 6, 24, and 48 h after treatments, immediately frozen in liquid nitrogen, and stored at −80°C until RNA extraction.

## Results

### Identification and sequence analysis of *14-3-3* gene family from *B. rapa*

The availability of initial draft of *B. rapa* genome project (http://brassicadb.org/brad/) led us to perform the comprehensive search of *14-3-3* gene sequences. Using *Arabidopsis* GRF (General Regulatory Factor) cDNA sequences as query, BLAST search in *B. rapa* genome database resulted in the identification of 21 putative gene sequences encoding 14-3-3 proteins (Table 1). The 21 *BraA.GRF14* genes were located on eight out of 10 linkage groups (LG) of *B. rapa* genome, with A07 LG containing up to six *BraA.GRF14* genes (Figure S1, Table 1). Adopting the standard gene nomenclature for *Brassica* species (Ostergaard and King, 2008), the genes were named as *BraA.GRF14.a* to *BraA.GRF14.u*, in order of their identification. The 21 *BraA.GRF14* genes were variable in their sizes ranging from 998 to 1857 bp. The coding DNA sequences (CDS) of *BraA.GRF14* genes ranged from 738 to 918 bp and shared 41.8–92.6% sequence identity among them (Table 1, Table S1).

**Table 1:** **Summary of gene structure attributes of the twenty-one 14-3-3 proteins identified in ***B. rapa*** genome (http://brassicadb.org/brad/)**.

**S. No**.	**Name**	**Gene ID in *B. rapa* database**	***B. rapa* linkage group**	**Gene size (bp)**	**CDS size (bp)**	**Protein size (aa)**	**No of exons**	**No of introns**	**Group**
1	*BraA.GRF14.a*	Bra031383	A5	1647	762	253	6	5	ε
2	*BraA.GRF14.b*	Bra024713	A9	1278	804	267	7	6	ε
3	*BraA.GRF14.c*	Bra016273	A8	1334	813	270	6	5	ε
4	*BraA.GRF14.d*	Bra012455	A7	1351	918	305	7	6	ε
5	*BraA.GRF14.e*	Bra035020	A7	1328	738	245	6	5	ε
6	*BraA.GRF14.f*	Bra000268	A3	1595	789	262	6	5	ε
7	*BraA.GRF14.g*	Bra035027	A7	1857	783	260	3	2	non-ε
8	*BraA.GRF14.h*	Bra008355	A2	1386	753	250	4	3	non-ε
9	*BraA.GRF14.i*	Bra003649	A7	1386	783	260	4	3	non-ε
10	*BraA.GRF14.j*	Bra016380	A8	1372	780	259	4	3	non-ε
11	*BraA.GRF14.k*	Bra028409	A7	998	777	258	4	3	non-ε
12	*BraA.GRF14.l*	Bra028187	A4	1029	774	257	4	3	non-ε
13	*BraA.GRF14.m*	Bra040592	A5	1082	786	261	4	3	non-ε
14	*BraA.GRF14.n*	Bra028586	A2	1730	828	275	4	3	non-ε
15	*BraA.GRF14.o*	Bra001049	A3	1053	786	261	4	3	non-ε
16	*BraA.GRF14.p*	Bra012325	A7	1207	792	263	4	3	non-ε
17	*BraA.GRF14.q*	Bra006061	A3	1382	756	251	4	3	non-ε
18	*BraA.GRF14.r*	Bra028068	A9	1159	786	261	4	3	non-ε
19	*BraA.GRF14.s*	Bra034430	A5	1058	792	263	3	2	non-ε
20	*BraA.GRF14.t*	Bra037818	A9	1346	750	249	4	3	non-ε
21	*BraA.GRF14.u*	Bra024384	A6	1566	747	248	4	3	non-ε

**Figure 1:**
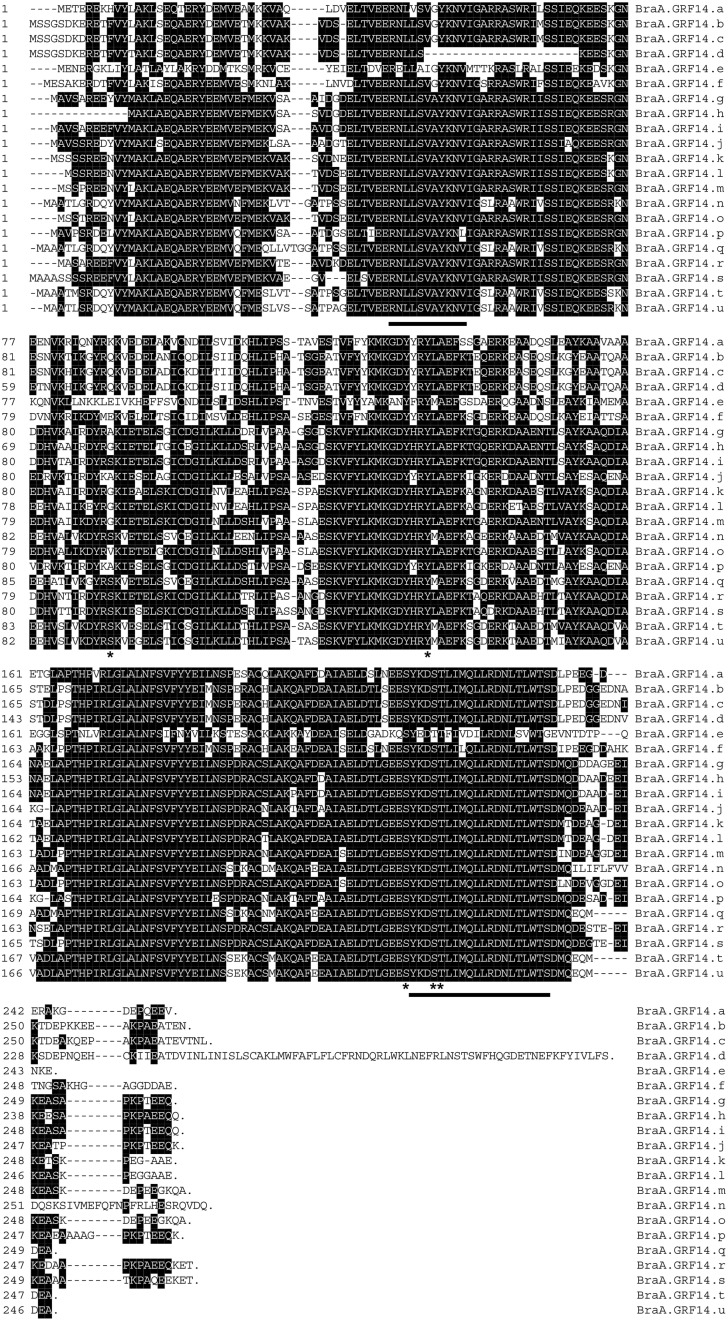
**Amino acid alignment of the deduced 14-3-3 proteins of ***B. rapa*** (BraA.GRF14)**. The sequence alignment of the 21 BraA.GRF14 proteins was performed using ClustalW. The 14-3-3 signature motifs RNL(L/V)SV(G/A)YKNV and YKDSTLIMQLLRDNLTLWTS, are underlined. The phosphorylation sites reported earlier for plant 14-3-3 proteins (Paul et al., 2012) are marked with asterisk.

The *BraA.GRF14* genes encoded proteins ranging from 245 to 305 amino acids, with their calculated molecular masses and pI that ranged from 27.79 to 35.28 kDa and 4.34 to 4.77, respectively. Amino-acid sequence alignment of the deduced BraA.GRF14 proteins indicated that these are highly conserved proteins (Figure 1) sharing a high level of sequence identity (45.4–96.8%) among them (Table S2). Further, when we queried the deduced BraA.GRF14 proteins at ExPASy proteomics tool (www.expasy.org) all proteins, except for BraA.GRF14.e (Bra035020), showed the presence of highly conserved 14-3-3 signature motifs RNL(L/V)SV(G/A)YKNV and YKDSTLIMQLLRDNLTLWTS, thereby confirming their identity as 14-3-3 proteins. The divergence observed in the deduced Bra035020 (BraA.GRF14.e) protein sequence might be an evolutionary consequence; although the possibility of its mis-annotation in the current version of *B. rapa* genome assembly cannot be ruled out completely. In addition, the conserved phosphorylation sites reported earlier for plant's 14-3-3 proteins were also present in BraA.GRF14 proteins. The pY137 of maize GF14-6 known to decreases binding of the H^+^-ATPase (Giacometti et al., 2004); and pS216, pS220, pT221 reported to be phosphorylated during the seed development in oilseed rape (Agrawal and Thelen, 2006), were all found to be conserved across BraA.GRF14 proteins. The pS93/95 residue, identified as being phosphorylated by SnRK2.8 in roots of the three *Arabidopsis* GRFs (χ, κ, ψ; Shin et al., 2007), was however found to be present in 7 of the 21 BraA.GRF14 proteins, thereby suggesting isoform-specific phosphorylation pattern and functional specificity of 14-3-3 proteins in *B. rapa*.

### Genomic structure and phylogenetic relationships of *B. rapa 14-3-3* genes

**Figure 2:**
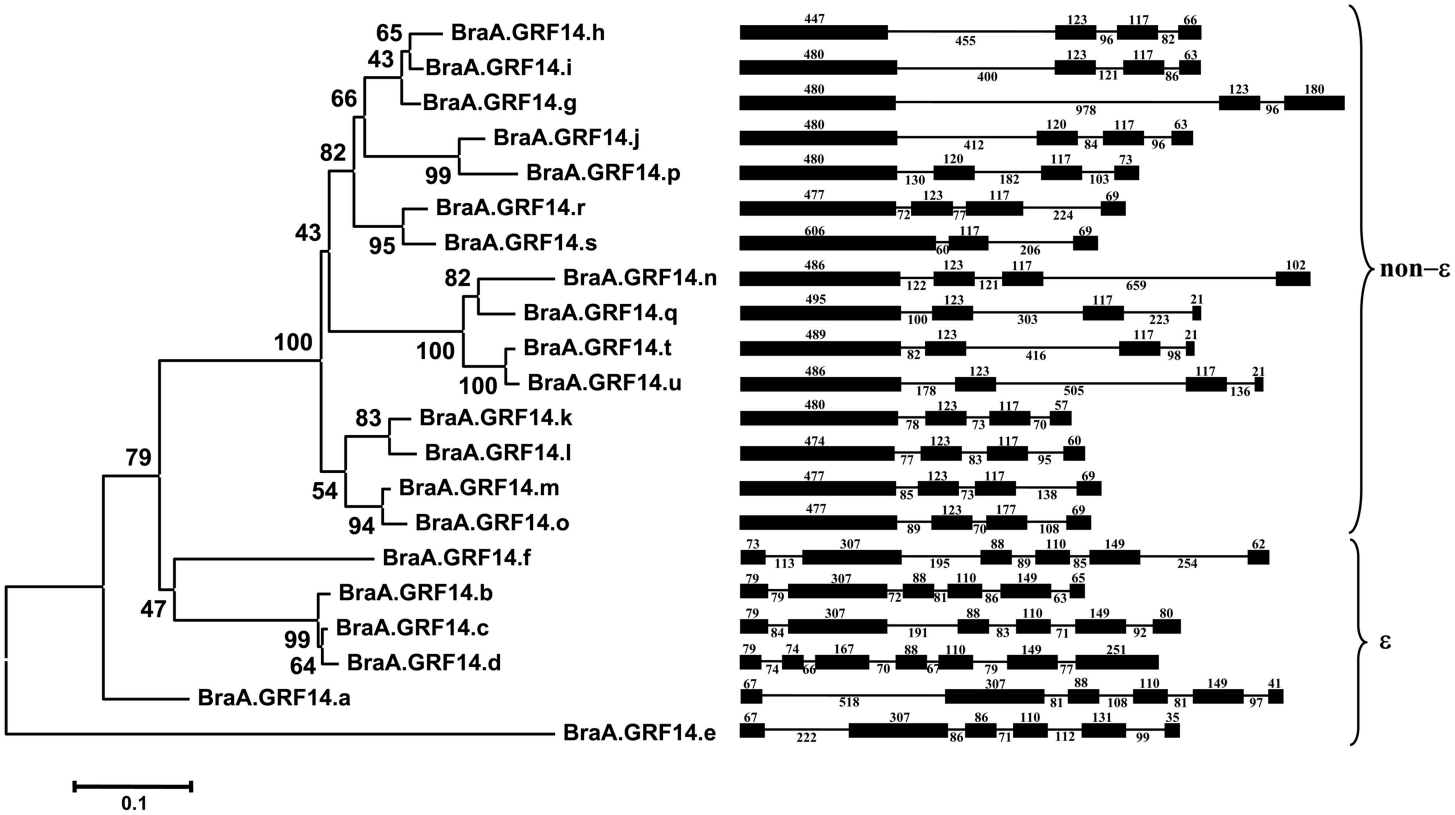
**Phylogenetic analysis and gene structures of the deduced 14-3-3 proteins of ***B. rapa*** (BraA.GRF14)**. The evolutionary history was inferred by using the Maximum Likelihood method based on the JTT matrix-based model conducted in MEGA5 (Tamura et al., 2011). The percentage of replicate trees in which the associated taxa clustered together in the bootstrap test (1000 replicates) is shown next to the branches. The tree is drawn to scale, with branch lengths measured in the number of substitutions per site. The sizes (in bp) and organization of exons (dark boxes) and introns (lines) of *BraA.GRF14* genes are marked along with.

The presence of multiple *14-3-3* type sequences in *B. rapa* having significant sequence divergence led us to investigate the evolution of *BraA.GRF14* gene family. We therefore, analyzed the genomic structure and phylogenetic relationship of *BraA.GRF14* gene family members. Analysis of genomic structure of 21 *BraA.GRF14* genes showed that the members of *14-3-3* gene in *B. rapa* contain 3–7 exons, interspersed by highly divergent introns (Figure 2). On the basis of organization of exons and introns, the *BraA.GRF14* genes were broadly categorized into two distinct sub-groups namely, the ε (epsilon) and non-ε (non-epsilon) groups. The six genes belonging to ε group contained 6–7 exons each, whereas 3–4 exons were present in the 15 non-ε group genes. Furthermore, the length of the exons was highly conserved among most of the 15 non-ε group genes; whereas genes belonging to ε group showed comparably higher divergence in their exon length. The extreme conservation of the exon organization in both ε and non-ε *BraA.GRF14* genes possibly suggest independent evolution and expansion of the ε and non-ε groups genes in *B. rapa*. In comparison, the introns in both ε and non-ε groups genes of *B. rapa* were highly divergent in their sizes and sequences.

To get a better insight into expansion of *B. rapa 14-3-3* gene family, a phylogenetic tree was constructed based on the multiple sequence alignment of full-length protein sequences from *B. rapa* (21), *Arabidopsis* (13) and *Oryza sativa* (7). Phylogenetic analysis showed that 14-3-3 protein isoforms from the three plant genomes were clustered into distinct ε and non-ε groups (Figure 2, Figure S2). Within each class, the 14-3-3 proteins from *Arabidopsis* and *B. rapa* genomes were grouped together, whereas the rice 14-3-3 proteins formed separate branches. This indicates that the 14-3-3 proteins of each class existed before the divergence of monocots and dicots, and have expanded independently in species-specific manner, as also observed for other gene families in plants (Zhang et al., 2005; Jain et al., 2007).

### Sub-genomic location and divergence analysis of *B. rapa 14-3-3* genes

It is well-known that the whole genome triplication (WGT) event in *Brassica* lineage had formed multiple homologs (paralogs) in each *Brassica* species. To analyze the degree of expansion of *14-3-3* genes between *B. rapa* and its nearest model plant *Arabidopsis*, we identify the sub-genomic location of the 21 *BraA.GRF14* genes on *B. rapa* genome (http://brassicadb.org/brad/). The *BraA.GRF14* genes were identified in all the three sub-genomes of *B. rapa*, with both least fractionated (LF) and most-fractionated (MF2) sub-genomes containing eight genes each, and the moderately fractionated (MF1) sub-genome having five genes only (Table 2).

**Table 2:** **Gene fractionation and divergence analysis of syntenic ***14-3-3*** genes identified in the three sub-genomes of ***B. rapa*** (http://brassicadb.org/brad/) with their corresponding ***Arabidopsis*** orthologs**.

**14-3-3 protein class**	**Gene**	**Protein name**	**Least fractionated sub-genome (LF)**	**Moderately fractionated sub-genome (MF1)**	**Most fractionated sub-genome (MF2)**
non-ε	*AtGRF1*	Chi	–	–	–
	*AtGRF2*	Omega	*BraA.GRF14.g* (0.81; 26.84)	*BraA.GRF14.h* (0.87; 29.12)	*BraA.GRF14.i* (0.57; 19.12)
				*BraA.GRF14.j* (0.99; 32.84)^*^	*BraA.GRF14.p* (0.94; 31.40)^*^
	*AtGRF3*	Psi	*BraA.GRF14.l* (0.70; 23.33)	*BraA.GRF14.k* (0.73; 24.34)	–
	*AtGRF4*	Phi	*BraA.GRF14.r* (0.78; 25.84)	–	*BraA.GRF14.s* (0.78; 26.15)
	*AtGRF5*	Upsilon	–	–	–
	*AtGRF6*	Lambda	–	*BraA.GRF14.q* (0.82; 27.37)	*BraA.GRF14.n* (0.98; 32.63)
	*AtGRF7*	Nu	*BraA.GRF14.m* (0.66; 21.93)	–	*BraA.GRF14.o* (0.48; 15.92)
	*AtGRF8*	Kappa	*BraA.GRF14.u* (0.90; 29.94)	–	*BraA.GRF14.t* (0.85; 28.23)
ε	*AtGRF9*	Mu	–	–	*BraA.GRF14.f* (0.45; 15.04)
	*AtGRF10*	Epsilon	*BraA.GRF14.a* (0.41; 13.82)	–	–
	*AtGRF11*	Omicron	–	–	–
	*AtGRF12*	Iota	*BraA.GRF14.b* (0.35; 11.56)	*BraA.GRF14.c* (0.32; 10.62)	*BraA.GRF14.d* (0.38; 12.71)
	*AtGRF13*	*Pi*	*BraA.GRF14.e* (0.45; 14.98)	–	–

*The Ks (synonymous substitution) and divergence time (mya, million years ago) of a B. rapa gene with its syntenic Arabidopsis ortholog, are given within parenthesis*.

* *BRAD database assign these as non-syntenic homologs of AtGRF10*.

Of the 13 *AtGRF14* genes, syntenic homologs for 10 could be identified in the *B. rapa*, with each *Arabidopsis* gene having variable number of *B. rapa* paralogs (Table 2; Figure S2). A total of five and three *B. rapa* paralogs was identified for *AtGRF2* (omega) and *AtGRF12* (iota) genes, respectively; whereas two paralogs were identified for five *Arabidopsis 14-3-3* genes in *B. rapa* genome. Only one *B. rapa* homologs was identified for three *AtGRF* genes. The gene-fractionation events in *Brassica* lineage in all possibility have led to the presence of variable number of *14-3-3* gene orthologs in *B. rapa*. Surprisingly, for three *14-3-3* genes of *Arabidopsis*, namely *AtGRF1, AtGRF5*, and *AtGRF11* no syntenic homolog could be identified in the *B. rapa* genome. Phylogenetic analysis showed that *AtGRF1/AtGRF4, AtGRF5/AtGRF7*, and *AtGRF11/AtGRF12* gene pairs formed individual branches, and could have arisen as the result of *Arabidopsis* specific At-α WGD event, dating around 24–40 mya (Franzke et al., 2011). Divergence analysis of these proteins pairs also showed that at least *AtGRF1/AtGRF4*, and *AtGRF5/AtGRF7* gene pairs might have duplicated recently in *Arabidopsis* lineage around 20.92 and 24.76 mya, respectively, after the *Arabidopsis–Brassica* split.

We further estimated the divergence of *BraA.GRF14* genes retained in the extant *B. rapa* genome by performing the pairwise comparisons to estimate the synonymous base substitution (*Ks*)-values between the duplicated *B. rapa* and *Arabidopsis* genes. Divergence times were calculated assuming a mutation rate of 1.5 × 10^−8^ synonymous substitutions per year (Koch et al., 2000). The *Ka/Ks* ratios of *Arabidopsis–B. rapa 14-3-3* orthologs were less than 1, suggesting purifying selection on these duplicated pairs (Table 2; Table S3). The *Ks*-values of *BraA.GRF14* genes ranged from 0.32 to 0.99, which indicated that the *BraA.GRF14* genes might have diverged somewhere between 10.62 and 32.84 million years ago (mya). Interestingly, the ε protein orthologs shared between the *Arabidopsis*–*B. rapa* genomes showed lower range of *Ks*-values (0.32–0.45) compared to the non-ε protein orthologs with higher range of *Ks*-values (0.48–0.99). This class-specific divergence pattern indicated that the non-ε proteins might have diverge recently (10.62–15.04 mya) compared to the ε proteins (>15.92 mya) during the evolution of extant *B. rapa* genome.

### Tissue specific expression of *BraA.GRF14* genes

The study on gene expression patterns of all the members of a gene family provides insight about their functional diversification. The multiplicity of *14-3-3* genes, therefore led us to investigate if all the 21 *BraA.GRF14* genes are expressed in *B. rapa*. Gene specific primers (Table S4) were designed for each *BraA.GRF14* genes and real time quantitative PCR (qRT-PCR) was performed using the cDNA samples prepared from different tissue types representing various stages of plant development.

It is interesting to note that the genes belonging to ε (epsilon) group, in general, showed lower levels of transcripts abundance in all the tissue type tested. For example, only two genes namely *BraA.GRF14.a* and *BraA.GRF14.f* showed moderate transcript abundance, when compared to the expression of *BraGAPDH*, whereas four genes namely *BraA.GRF14.b, BraA.GRF14.c, BraA.GRF14.d*, and *BraA.GRF14.r* showed almost negligible transcript abundance across different tissue types tested. In contrast, among the 15 non-ε group genes, only three genes (*BraA.GRF14.n, BraA.GRF14.s*, and *BraA.GRF14.u*) showed lower transcript accumulation across plant development. This observation was confirmed using multiple primer pairs from different regions of the representative genes (Table S4). The quantitatively higher transcript abundance of non-ε group genes compared to the ε group genes obtained for *B. rapa 14-3-3* gene family, is somewhat similar to the expression observed for *AtGRF* genes in *Arabidopsis* (Table S5).

**Figure 3:**
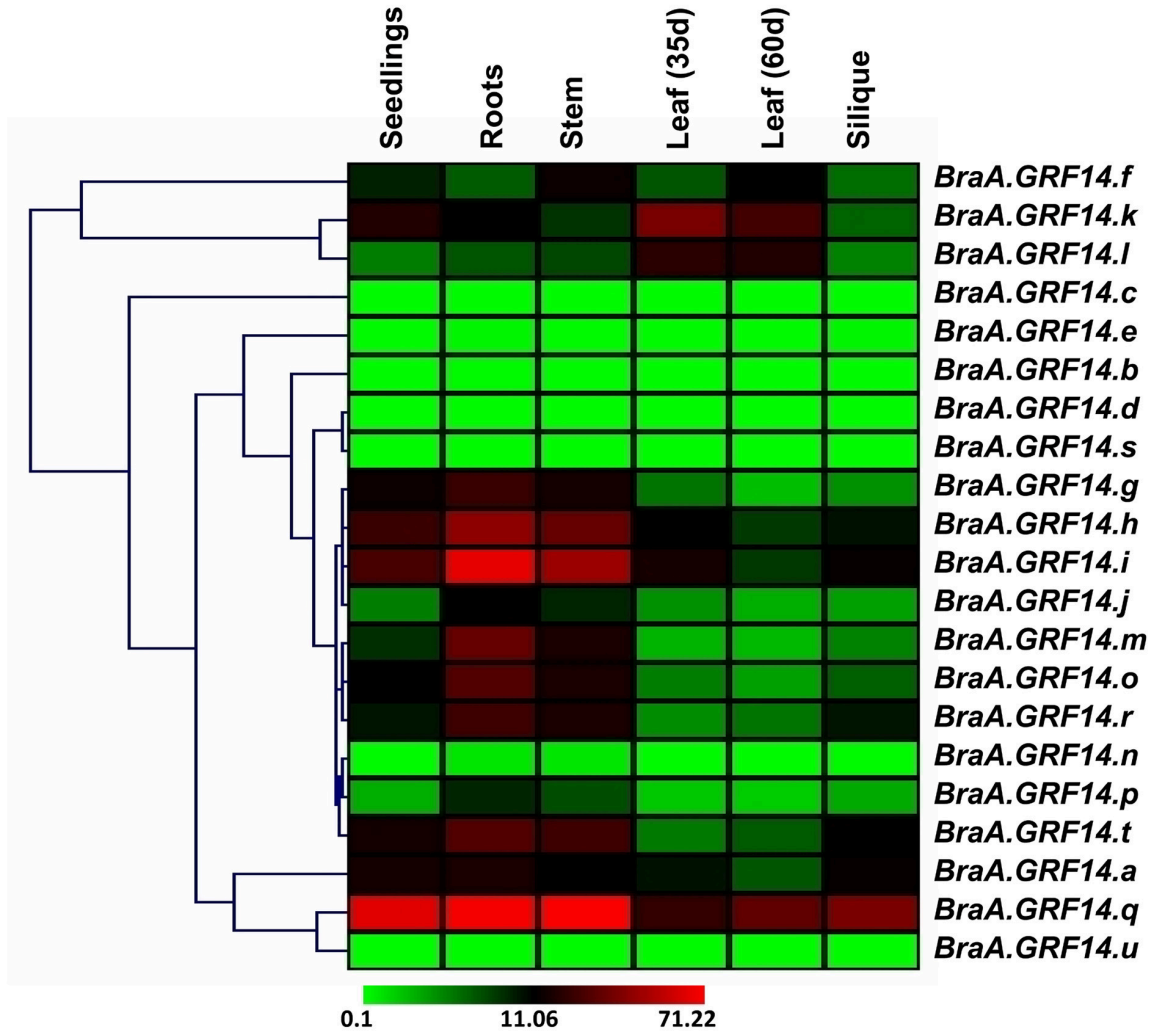
**Expression profile of ***B. rapa 14-3-3*** genes (***BraA.GRF14***) across plant developmental stages**. A heat map representing hierarchical clustering of average fold values of *BraA.GRF14* genes expression (w.r.t. *GAPDH*, set at 100) in various developmental tissues (mentioned at the top of each lane) is shown. The color scale representing average signal is shown at the bottom of the heat map.

The *BraA.GRF14* genes also exhibited a high degree of tissue specificity (Figure 3). Hierarchal clustering based on gene expression profile suggested that *BraA.GRF14* genes, in general, are abundantly expressed in root, stem and seedling stages, compared to leaf and silique stages where moderate level of transcript abundance was detected. In contrast, two of the genes namely, *BraA.GRF14.k* and *BraA.GRF14.l* showed a high level of transcript abundance in the developing leaf stages only, thereby suggesting that these members may play a specialized role in these tissue types.

We also examined the expression pattern of multiple paralogs of each *GRF* gene, resulted from WGT event in *Brassica* lineage. Hierarchical clustering of three paralogs of *GRF2* namely *BraA.GRF14.g, BraA.GRF14.h*, and *BraA.GRF14.i* showed almost similar expression levels and tissue specificity when tested across different tissue types of *B. rapa* (Figure 3). The *B. rapa* paralogs of *GRF3* (*BraA.GRF14.k* and *BraA.GRF14.l*) and *GRF7* (*BraA.GRF14.m* and *BraA.GRF14.o*) also showed similar expression patterns, thereby suggesting that these *B. rapa* paralogs, resulted from polyploidization, had conserved their expression levels and tissue-specificity during the evolution of *Brassica* species. However, in other cases the paralogs of *GRF4* (*BraA.GRF14.r* and *BraA.GRF14.s*), *GRF6* (*BraA.GRF14.q* and *BraA.GRF14.n)*, and *GRF8* (*BraA.GRF14.u* and *BraA.GRF14.t*), showed contrasting variation in their transcript abundance in different tissue type tested, suggesting tissue-specific transcriptional sub-functionalization of *B. rapa 14-3-3* paralogs.

### Expression analysis of *14-3-3* genes in response to abiotic stresses and hormone treatments in *B. rapa*

To investigate the effects of various abiotic stress conditions and hormone treatments on the expression of *BraA.GRF14* genes, *B. rapa* seedlings were treated with different abiotic stress conditions (dehydration, cold, heat, salt) and exogenously supplied hormones (IAA, MeJA, SA, and ABA) for different time points (15 min, 30 min, 3 h, and 6 h). The qRT-PCR expression analysis was performed to detect the transcriptional regulation of 14 *BraA.GRF14* genes, having detectable transcripts levels, compared to the untreated control seedlings.

**Figure 4:**
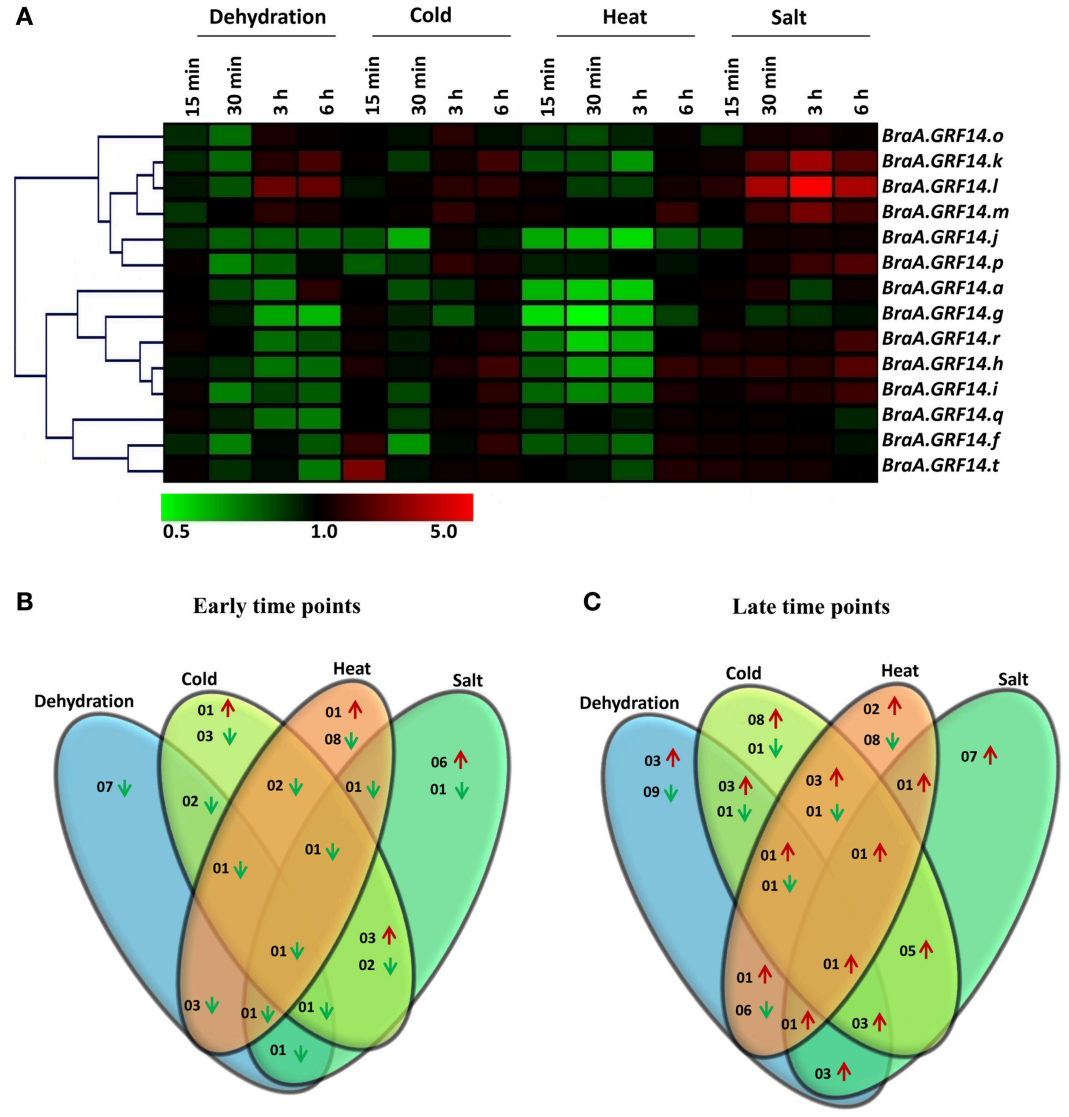
**Expression profile of ***BraA.GRF14*** genes in 6 days old ***B. rapa*** seedlings in response to abiotic stress conditions**. **(A)** A heat map representing hierarchical clustering of average fold change of *BraA.GRF14* genes expression in response to dehydration, cold (4°C), heat (37°C), and salt (300 mM NaCl) treatments at different time points (15 min, 30 min, 3 h, and 6 h) is shown (mentioned at the top of each lane). The mock treated seedlings of same time interval served as control (set at 1) and expression was normalized with *ACT2* gene. The color scale representing average signal is shown at the bottom of the heat map. The Venn diagrams represent the total number of *BraA.GRF14* genes which were upregulated (red upward arrow) and down-regulated (green downward arrow) during **(B)** early (15 and 30 min), and **(C)** late (3 and 6 h) abiotic stress conditions.

The expression of *BraA.GRF14* genes were altered differentially in response to different abiotic stress treatments (Figure 4A). The expression of *BraA.GRF14* genes was found to be unaltered or down-regulated, particularly during the early stages (15 and 30 min) of dehydration and heat stress treatments compared to higher induction observed during later time points (3 and 6 h; Figures 4B,C). Most of the *BraA.GRF14* genes were found to be highly induced by salt treatment at all tested time points, thereby suggesting their crucial roles during salt stress conditions (Figure 4A). In response to cold, *BraA.GRF14.t* was up-regulated within 15 min, whereas transcript accumulation of eight *BraA.GRF14* genes was found to be induced after delayed cold treatment. In response to dehydration, none of the *BraA.GRF14* genes were up-regulated during early time points, whereas three *BraA.GRF14* genes namely, *BraA.GRF14.k, BraA.GRF14.l*, and *BraA.GRF14.m* showed up-regulation in their transcripts during later time points (Figures 4B,C). Under cold and salt treatments, five *BraA.GRF14* genes namely, *BraA.GRF14.h, BraA.GRF14.i, BraA.GRF14.k, BraA.GRF14.l*, and *BraA.GRF14.m*, were found to be commonly induced during late time points (Figures 4A,C). Similarly, the three common genes namely *BraA.GRF14.k, BraA.GRF14.l*, and *BraA.GRF14.m* were also found to be up-regulated during the later time points of cold, dehydration and salt stresses. However, using the stringent criterion, expression of only one *BraA.GRF14* gene i.e., *BraA.GRF14.m* was found to be up-regulated during late time points under all the four tested abiotic stress conditions (Figure 4C).

**Figure 5:**
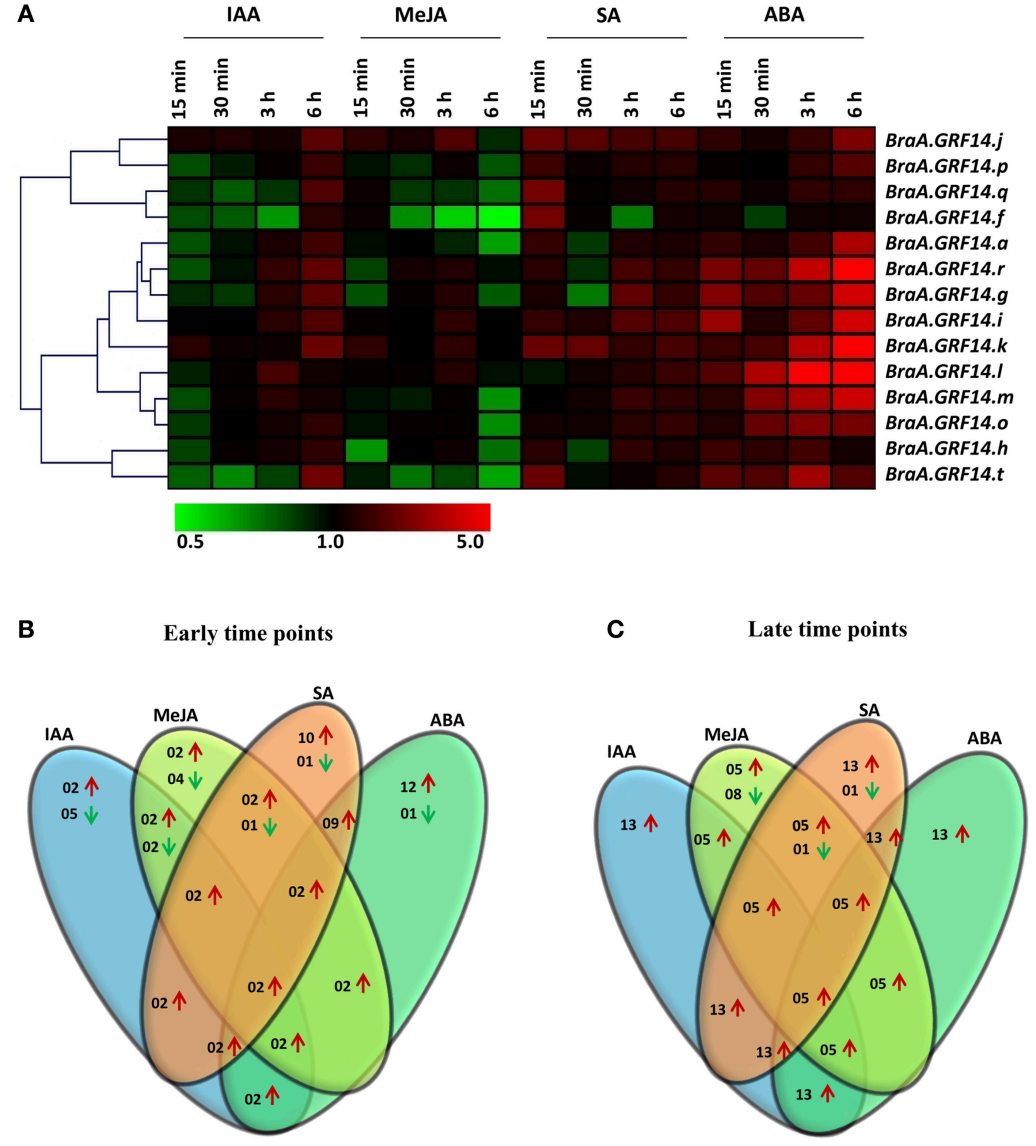
**Expression profile of ***BraA.GRF14*** genes in 6 days old ***B. rapa*** seedlings in response to phytohormone treatments**. **(A)** A heat map representing hierarchical clustering of average fold change of *BraA.GRF14* genes expression in response to methyl jasmonate (MeJA, 0.2 mM), salicylic acid (SA, 0.2 mM), 1-aminocyclopropane carboxylate (ACC, 0.1 mM), indole-3-acetic acid (IAA, 0.1 mM), and abscisic acid (ABA, 0.1 mM) at different time points (15, 30 min, 3 and 6 h) is shown (mentioned at the top of each lane). The mock treated seedlings of same time interval served as control (set at 1) and expression was normalized with *ACT2* gene. The color scale representing average signal is shown at the bottom of the heat map. The Venn diagrams represent the total number of *BraA.GRF14* genes which were upregulated (red upward arrow) and down-regulated (green downward arrow) during **(B)** early (15 and 30 min), and **(C)** late (3 and 6 h) phytohormone treatments.

The transcriptional regulation of *BraA.GRF14* genes in response to exogenously supplied phytohormones was also analyzed. Our results showed that most of the *BraA.GRF14* genes were differentially expressed and showed up-regulation in their transcript particularly during later time points (3 and 6 h; Figures 5A–C). Of 14 *BraA.GRF14* genes, a total of 13, 5, 13, and 13 genes were found to be up-regulated during later time points on IAA, MeJA, SA, and ABA treatments, respectively, thereby suggesting that *B. rapa* 14-3-3 proteins could mediate various plant responses *via* phytohormone sensing and signaling. In response to IAA treatment, the *BraA.GRF14* genes showed up-regulation of their transcripts at a later time point (6 h); whereas the exogenous treatment of ABA showed a pronounced up-regulation of almost all the *BraA.GRF14* genes except *BraA.GRF14.f* within few minutes, suggesting differential transcriptional response of *B. rapa 14-3-3* genes during abiotic stress. In response to SA treatment, a hormone mimicking the biotic stress condition, the expression of most of the *BraA.GRF14* genes was found to be induced within 15 min and up to 6 h of treatment. On contrary to this, with the application of MeJA on *B. rapa* seedlings, the expression of only two *BraA.GRF14* genes namely, *BraA.GRF14.j* and *BraA.GRF14.k* showed up-regulation in their transcript during early time points (Figures 5A,B). In response to all the tested phytohormone treatments, although two genes (*BraA.GRF14.j* and *BraA.GRF14.k*) were found to be up-regulated during early time points; a total of five genes (*BraA.GRF14r; BraA.GRF14.i, BraA.GRF14.j, BraA.GRF14.k, and BraA.GRF14.l*) were up-regulated during later time points. None of the single *BraA.GRF14* gene was found to be commonly down-regulated in response to all the phytohormone treatments, during both early and late time points.

### Expression analysis of *14-3-3* genes during nutrient deprivation conditions in *B. rapa*

Previous findings suggest a connection of *14-3-3* isoforms with plant nutrient metabolism and signaling (Xu and Shi, 2006; Shin et al., 2011). To better understand the transcriptional regulation of *14-3-3* genes toward changes in plant nutrient status, *B. rapa* seedlings were deprived of nitrogen (N), phosphorus (P), and potassium (K) from early (1 and 6 h) to late (24 and 48 h) time intervals, and the expression of *BraA.GRF14* genes was analyzed using qRT-PCR.

**Figure 6:**
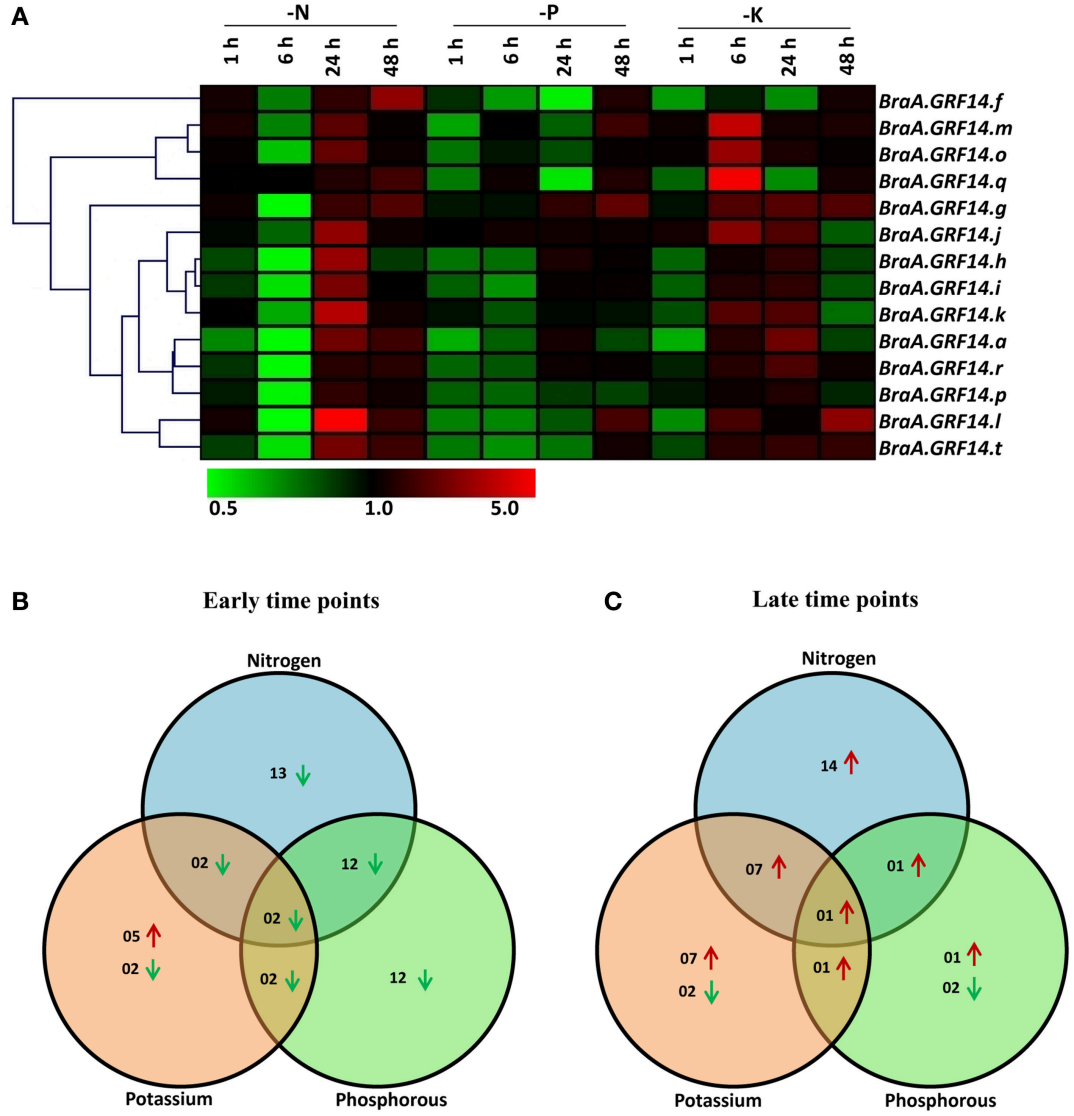
**Expression profile of ***BraA.GRF14*** genes during nutrient deprivation conditions in 5 days old ***B. rapa*** seedlings**. **(A)** A heat map representing hierarchical clustering of average fold change of *BraA.GRF14* gene expression in response to nutrient deprived conditions at different time points (mentioned at the top of each lane). The 5 days old *B. rapa* seedlings were harvested at different time points (1, 6, 24, and 48 h) after they were exposed to nutrient solution lacking Nitrogen (N), Phosphorus (P), and Potassium (K). The mock treated seedlings of same time interval served as control (set at 1) and expression was normalized with *ACT2* gene. The color scale representing average signal is shown at the bottom of the heat map. The Venn diagrams represent the total number of *BraA.GRF14* genes which were upregulated (red upward arrow) and down-regulated (green downward arrow) during **(B)** early (1 and 6 h), and **(C)** late (24 and 48 h) nutrient deprived conditions.

The *BraA.GRF14* genes showed differential transcriptional variation in response to the tested nutrient deprivation conditions. It was observed in case of nitrogen deprivation condition (-N), the transcript abundance of most of the *BraA.GRF14* isoforms was found to be down-regulated by less than two-folds at early time points (Figures 6A–C). Interestingly, during delayed nitrogen deprivation (24 and 48 h), the expression of almost all *BraA.GRF14* genes showed pronounced up-regulation. In response to phosphorus deprivation condition (-P), the expression of 12 *BraA.GRF14* genes were found to be down-regulated during early time points, whereas after delayed phosphorus deficiency only *BraA.GRF14.g* was found to be up-regulated (Figures 6B,C). In contrast, the potassium deficient (-K) *B. rapa* seedlings showed a profound up-regulation of most of the *BraA.GRF14* genes within 6 h of treatment (Figure 6A). For example, expression of *BraA.GRF14.g, BraA.GRF14.j, BraA.GRF14.m, BraA.GRF14.o*, and *BraA.GRF14.r* genes were significantly up-regulated during early time points. During the prolonged K deprivation condition, the expression of seven *BraA.GRF14* was also found to be up-regulated, suggesting a profound yet differential transcriptional response of *B. rapa 14-3-3* genes in response to -K condition (Figures 6B,C). Overall, in response to all the tested nutrient deprivation conditions, two genes namely, *BraA.GRF14.f* and *BraA.GRF14.h* were commonly down regulated during early time points; whereas *BraA.GRF14.g* was only found to be up-regulated during later time points (Figures 6B,C).

### Analysis of *cis*-regulatory divergence of 5′ upstream sequences of *B. rapa 14-3-3* genes

The differential transcriptional regulation of *BraA.GRF14* genes during plant growth and developmental stages and in response to various elicitor treatments tested in this study could be attributed to sequence divergence and *cis*-regulatory elements present in their 5′ upstream regulatory sequences. Approximately 1.5 kb sequence upstream of transcription start site of *BraA.GRF14* genes were obtained from the BRAD database and analyzed using ClustalW. The 5′ upstream sequence of *BraA.GRF14* genes were highly divergent showing a low level of sequence identity ranging from 18.5 to 31.7% (Table S6), which is quite in agreement with the differential expression pattern obtained among *BraA.GRF14* genes.

To identify various *cis*-acting regulatory elements present in the 5′ upstream sequences, the *BraA.GRF14* genes were further analyzed using the PLACE database (http://www.dna.affrc.go.jp/PLACE/). *In-silico* analysis revealed the presence of various motifs involved in phytohormones, abiotic and biotic stress responses (Table S7). In general, the *BraA.GRF14* genes, which were found to be up-regulated under ABA treatment in current study, showed abundance of ABA responsive elements like ABRELATERD1, DRE1COREZMRAB17, and ABREZMRAB28, thereby indicating the involvement of *B. rapa 14-3-3* gene family members in various ABA mediated cellular responses. Similarly, upstream sequence of *B. rapa 14-3-3* genes like *BraA.GRF14.g, BraA.GRF14.h, BraA.GRF14.p*, and *BraA.GRF14.m*, which were significantly up-regulated under salt stress, were found to have GT1GMSCAM4 response element known to be involved in salt stress and plant defense. Various pathogen and elicitor response elements like ELRECOREPCRP1, GCCCORE, T/GBOXATPIN2, and TATCCACHVAL21 were also present, which confirm up-regulation of few *BraA.GRF14* genes under MeJA and SA treatments. A significant up-regulation of most of the *BraA.GRF14* genes during late time point of IAA treatment (6 h) can also be correlated with the presence of different types of auxin inducible *cis*-acting elements like ARFAT, CATATGGMSAUR, D3GMAUX28, and NTBBF1ARROLB in their 5′ upstream sequences. Expression of all the *B. rapa 14-3-3* genes were either unaltered or down-regulated during different time point of heat stress, which could be due to the presence of only few heat stress related *cis*-acting elements. Although the upstream sequence of *B. rapa 14-3-3* genes showed the presence of few *cis*-acting elements for nutrient deprivation conditions, but interestingly all the *B. rapa 14-3-3* genes were highly expressed during late time of nutrient deprivation, which could be due to the presence of still unknown regulatory motifs involved in the nutrient sensing in *B. rapa*. Nonetheless, the differential transcriptional alteration observed in the expression of *B. rapa 14-3-3* genes could be nicely attributed to the presence of various *cis*-regulatory elements and their quantitative variability, across multiple *BraA.GRF14* genes.

## Discussion

The 14-3-3 proteins are a family of highly conserved regulatory proteins present across phyla, which function by binding to the phosphorylated target proteins (effectors) to play vital roles in many biological processes in plants, including primary metabolism and hormone signaling as well as in response to the abiotic and biotic stresses. In this study, through data mining we identified 21 genes encoding 14-3-3 like proteins from the recently sequenced *B. rapa*, the model *Brassica* genome.

### Evolutionary expansion of *B. rapa 14-3-3* multigene family

It is quite expected that the inherent polyploidy in plants has shaped the expansion of *14-3-3* gene family. For example, Li and Dhaubhadel (2011) identified 18 genes encoding 14-3-3 proteins in soybean, an allotetraploid genome (ca. 1115 Mb), having undergone two whole genome duplication events (ca. 14 and 42 mya). Comparative genomics study in cotton, an allotetraploid crop species (ca. 2500 Mb), identified the highest thirty-one *14-3-3* cDNAs encoding 25 unique proteins, resulting from a recent duplication event after the divergence of cotton from its progenitor species (Sun et al., 2011).

It is interesting that given the higher size (haploid genome ca. 500 Mb) and ploidy (mesohexaploidy) level of the *B. rapa* genome compared to that of the diploid *A. thaliana* (ca. 120 Mb), the *B. rapa* has most likely only 21 isoforms of 14-3-3 proteins, in comparison with 13 expressed isoforms reported in the closest dicot model (Figure 1). Various comparative genomics studies have clearly suggested that the *Arabidopsis* and the cultivable *Brassica* species had split from a common ancestral Brassicaceae around 13–17 million years ago (mya; Lysak et al., 2007; Panjabi et al., 2008; Franzke et al., 2011; Wang et al., 2011; Cheng et al., 2013). The *Brassica* lineage has further undergone whole genome triplication (WGT) event after the *Arabidopsis*–*Brassica* split, as a result of which the so called diploid *Brassica* species, including *B. rapa*, are paleohexaploid containing three sub-genomes. Sequence level studies in recent year strongly suggested that among these three sub-genomes, biased gene-fractionation (gene-loss) phenomenon has occurred, which resulted in the formation of least fractionized (LF), moderate gene fractionized (MF1), and the most gene fractionized (MF2) sub-genomes in *B. rapa* (Cheng et al., 2013). Thus, all *Brassica* species analyzed to date are supposed to contain multiple copies of orthologous genomic regions of *A. thaliana* (Lysak et al., 2007; Panjabi et al., 2008). We presume that the variable copies (1–5) of each *14-3-3 Arabidopsis* genes identified in *B. rapa* could be the consequence of differential level of gene-fractionation (or gene-loss) phenomenon that occurred after the WGT event in the extant *B. rapa* genome (Table 2). As extreme cases, for few *Arabidopsis 14-3-3* genes no syntenic ortholog were observed in *B. rapa*. Nonetheless, polyploidy coupled with genomic shrinkage and rearrangements have caused noteworthy expansion of *14-3-3* gene family members (21 isoforms) in mesohexaploid *B. rapa*, compared to the *A. thaliana*, rice, soybean, and cotton having 13, 8, 18, and 25 members, respectively.

Sequence alignment revealed that the orthologous genes shared between *Arabidopsis–B. rapa* genomes have a high level of amino-acid similarity, suggesting possibility of functional conservation of the 14-3-3 proteins across the two genomes. The *B. rapa* 14-3-3 proteins could be classified into two groups namely epsilon (ε) and non-epsilon (non-ε), with each sub-group showing extreme conservation of the intron-exon organization, and a distinct divergence pattern (*Ks*-values) between the two sub-groups, which in all possibility suggest independent evolution and expansion of the ε and non-ε groups *14-3-3* genes in *B. rapa*. Such class specific divergence pattern is also reported recently for the three types of plant G-gamma (Gγ) subunits (Type-A, -B, and -C) of the heterotrimeric G-proteins (Trusov et al., 2012; Arya et al., 2014; Kumar et al., 2014), an important class of signaling proteins, wherein the divergent Gγ subunits are known to provide functional selectivity to G-proteins in plants. We presume that the quantitative variation as well as the divergent residues present across *14-3-3* isoforms could shape some degree of specificity with regard to their expression profiles and the target protein(s) with which they interact, thereby contributing to the remarkable phenotypic plasticity and environmental adaptability of *B. rapa*.

### *B. rapa 14-3-3* genes exhibit redundant and divergent expression patterns

Gene duplication although raises the functional redundancy of duplicated genes, it is known to serve as a mechanism to increase the functional diversity. The duplicated genes often evolved *cis*-regulatory divergence in their regulatory regions; as a consequence there exist both immediate and long-term alterations in the expression of genes arising from polyploidy, such as differential expression, transcriptional bias, or gene silencing (Adams, 2007; Chaudhary et al., 2009). As a result, the duplicated genes may undergo diversification of gene function(s) such as neo-, sub- or, non-functionalization.

The *BraA.GRF14* genes are ubiquitously expressed during *B. rapa* growth and developmental stages (Figure 3). Since 14-3-3 proteins represent one of the key components of the plant signaling cascade (reviewed in Sehnke et al., 2002; de Boer et al., 2013), the ubiquitous activity of BraA.GRF14 proteins *vis-à-vis* their interaction with their client proteins, are quite necessary for regulating a wide variety of biological processes in *B. rapa*. Interestingly, the class specific transcription abundance of the *B. rapa* non-ε group genes compared to the ε group genes observed in this study, is somewhat similar to that of the *Arabidopsis 14-3-3* genes (Paul et al., 2012). Three of the five ε group genes of *Arabidopsis* have significantly low expression intensities compared to the highly expressed non-ε group genes (Table S4). This evolutionary conservation of expression pattern of the *Arabidopsis–Brassica* orthologs in all possibility suggests that non-ε group *14-3-3* genes might have retained functional dominance in regulating various growth and development processes across Brassicaceae.

Recent studies in polyploid *Brassica* species have functionally demonstrated that members of a multigene family can be expressed at different levels and can respond differentially to polyploidy in various organs of the plant or in response to various environmental stimuli (Higgins et al., 2012; Augustine et al., 2013; Meenu et al., 2015). Our study also suggest that multiple paralogs of few *GRF* genes, resulted from WGT event in *Brassica* lineage, although show almost similar expression patterns and tissue specificity; whereas in other cases the paralogs have contrasting variation in their transcript abundance and pattern across plant developmental stages. The overlapping and divergent expression patterns of *BraA.GRF14* genes suggest that the multiple members have evolved to perform redundant yet tissue-specific functions during plant growth and development in *B. rapa*.

### *B. rapa 14-3-3* genes are differentially regulated in response to various stimuli

There are evidences that plant 14-3-3 proteins show changes in their gene expression in response to various environmental stresses (Roberts et al., 2002). These stresses often have variable effects on different isoforms in terms of change in expression level and the time period. Our study also demonstrates that the expressed *B. rapa 14-3-3* genes behave differentially in response to the tested abiotic stress conditions, exogenously supplied phytohormones, and nutrient deprivation conditions indicating that different members of this gene family might have undergone differential transcriptional regulation to play specific roles during altered environmental conditions.

In general, most of the *BraA.GRF14* genes were significantly induced during high salt treatment both during early and late time points (Figure 4). The rice *OsGF14f* was originally identified as the *14-3-3* transcript that accumulated in the callus and seedling of rice when exposed to high salt or cold temperature (Kidou et al., 1993). Similarly, the up-regulation of *14-3-3* genes has been reported earlier under NaCl treatment in tomato, cotton and rice (Chen et al., 2006; Xu and Shi, 2006; Yao et al., 2007; Sun et al., 2011). In *Arabidopsis*, two *14-3-3* genes namely, *RCI1A* and *RCI1B* were shown to be involved in cold and freezing stress tolerance (Jarillo et al., 1994; Catalá et al., 2014). Under cold stress condition, eight *BraA.GRF14* genes were up-regulated during later time points (Figure 4C). Likewise, the expression of few members of *Phaseolus vulgaris 14-3-3* gene family has been recently reported to be up-regulated by cold stress (Li et al., 2015). Over-expression of the *Arabidopsis 14-3-3* “*lambda*” gene into cotton has been shown to impart enhanced tolerance to drought in transgenic lines, as determined by less wilting and visible damage to the leaves (Yan et al., 2004). Interestingly, we found only few *BraA.GRF14* genes were up-regulated under dehydration and heat stress both during early and later time points (Figure 4), suggesting that *BraA.GRF14* gene members have evolved to perform condition-specific functions.

It has been widely accepted that *14-3-3* genes act as key components in regulating phytohormone mediated plants responses (reviewed in Denison et al., 2011). Our study shows a profound up-regulation of *BraA.GRF14* genes under MeJA and SA treatments during later time points (Figure 5), indicating that *B. rapa 14-3-3* multigene family plays regulatory roles in response to biotic stress. Earlier, it has been shown that *14-3-3* genes are involved in plant defense response in poplar (Lapointe et al., 2001). Similarly, altered expression pattern of *14-3-3* genes under various biotic stress conditions has been reported in rice (Chen et al., 2006; Yao et al., 2007). The constitutive up-regulation of *BraA.GRF14* genes under ABA treatment, coupled with the presence of high number of ABA responsive *cis*-regulatory elements in their 5′ upstream region, suggests that the *B. rapa* 14-3-3 proteins play a vital role in ABA mediated cellular responses. The 14-3-3 proteins are also known to interact with the ABA signaling pathway in barley, cotton and rice, by interacting with the AREB-like transcription factors (ABF1, ABF2, ABF3, and ABI5), that binds to ABA-responsive elements (Schoonheim et al., 2007; Zhang et al., 2010; Hong et al., 2011). The differential transcriptional regulation of *BraA.GRF14* genes in response to the tested phytohormones suggests their complex cross-talk with phytohormone signaling components to regulate wide range of physiological processes.

In higher plants, 14-3-3 proteins play a significant role in response to nutrient sensing. The phosphorus deprivation (-P) condition, in general, causes a significant down-regulation of *B. rapa 14-3-3* gene expression (Figure 6). This is in agreement with that observed for *Arabidopsis GRF* orthologs (Cao et al., 2007), thereby suggesting that P deficiency potentially affects transcript levels of *14-3-3* isoforms and their dependent processes in different cellular compartments. In response to nitrogen deprivation (-N) condition, the expression of *B. rapa 14-3-3* genes was induced at later time points. In contrast, potassium deprivation (- K) treatment seems to up-regulate the expression of most of the *14-3-3* isoforms as also reported for tomato 14-3-3 proteins (Wang et al., 2002; Xu and Shi, 2006). The expression of *BraA.GRF14.t* gene was increased under potassium deprivation (-K) condition during the late time points. Similarly, the expression level of *Arabidopsis 14-3-3* κ increased after “K” deprivation in leaves (Shin et al., 2011). The plant's 14-3-3 proteins are known to interact with regulatory enzymes involved in nutrient sensing, metabolism and transport in plants. Glutamate synthase (GS) and nitrate reductase, key enzymes that regulate N metabolism, were identified as a 14-3-3 interacting proteins (Bachmann et al., 1996; Shin et al., 2011). Various signaling proteins including 14-3-3 interact with phosphorus deficiency response factors including protein kinases, phosphatases (Baldwin et al., 2008; Xu et al., 2012). Regulatory evidences of *14-3-3* genes were also observed of plant K^+^ channels, which are known to play a role in potassium homeostasis in the plants (Bunney et al., 2002; Wijngaard et al., 2005). The differential transcriptional response of *14-3-3* genes in response to abiotic stress conditions, phytohormones treatments, and nutrient deprivation conditions indicated that each member of this family participate for condition specific function in multiple signaling pathways.

It is well-known that various post-translational modifications of proteins increase the functional diversity of the proteome by the covalent addition of functional groups or proteins, proteolytic cleavage of regulatory subunits or degradation of the entire protein. Recent studies in *Arabidopsis* and other plants suggested evidences for phosphorylation of several isoforms at conserved as well as divergent serine (S) and tyrosine (Y) residues, providing the potential for phosphorylation to affect 14-3-3 proteins in an isoform-specific manner (reviewed by Paul et al., 2012). Thus, in addition to the transcriptional regulation observed in the current study, the isoform-specific phosphorylation patterns could also provide a key post-translational regulation of the multiple BraGRF14 proteins toward controlling their binding with “client” proteins and the functional diversity in *B. rapa*.

In conclusion, our study provides a comprehensive classification together with a structural and evolutionary analysis of the *BraA.GRF14* gene family in *B. rapa*. A total of 21 isoforms of *BraA.GRF14*s are identified from *B. rapa*, of which 14 isoforms were found to be expressed throughout the plant developmental tissues. The *BraA.GRF14* genes, on the basis of introns and exons, were broadly categorized into two distinct sub-groups namely the ε (epsilon) and non-ε (non-epsilon) groups. It was also observed that multiple orthologs for most of the *Arabidopsis 14-3-3* genes existed in *B. rapa*. Expression analysis of the 14 *BraA.GRF14* genes in response to abiotic stress, hormone stress and nutrient deficiency in the *B. rapa* seedlings suggest that *B. rapa* 14-3-3s are either directly or indirectly involved in the regulation of majority of physiological and metabolic pathways. A systemic and comparative analysis of the target proteins of the *B. rapa* 14-3-3 isoforms, in future, would contribute to fundamental understanding of the conservation and divergence of biological processes controlled by these key signaling proteins.

## Author contributions

RC, RA conducted the real time PCR experiments and data analysis. Kanchupati P, RK, Kumar P, GA carried out identification and *in-silico* analysis of candidate genes. RC, RK, and NB compiled the data and writing of manuscript. All authors read and approved the final manuscript.

## Funding

This work was supported by the core research grant from the NIPGR, India. RC and RA were supported from NIPGR short-term research fellowships; Junior Research Fellowships of CSIR (to GCA), UGC (to Kanchupati P, RK), and DBT (to Kumar P) are also acknowledged.

### Conflict of interest statement

The authors declare that the research was conducted in the absence of any commercial or financial relationships that could be construed as a potential conflict of interest. The reviewer Yashwanti Mudgil and handling Editor Girdhar Kumar Pandey declared their shared affiliation, and the handling Editor states that the process nevertheless met the standards of a fair and objective review.
