# Supplementary material for: Class-Specific Evolution and Transcriptional Differentiation of 14-3-3 Family Members in Mesohexaploid Brassica rapa
Source: Front Plant Sci. 2016 Jan 26;7:12. doi: 10.3389/fpls.2016.00012 (PMC4726770; doi:10.3389/fpls.2016.00012)
Supplement: Supplementary file 8 [file Presentation1.PPT]

## Slide 1
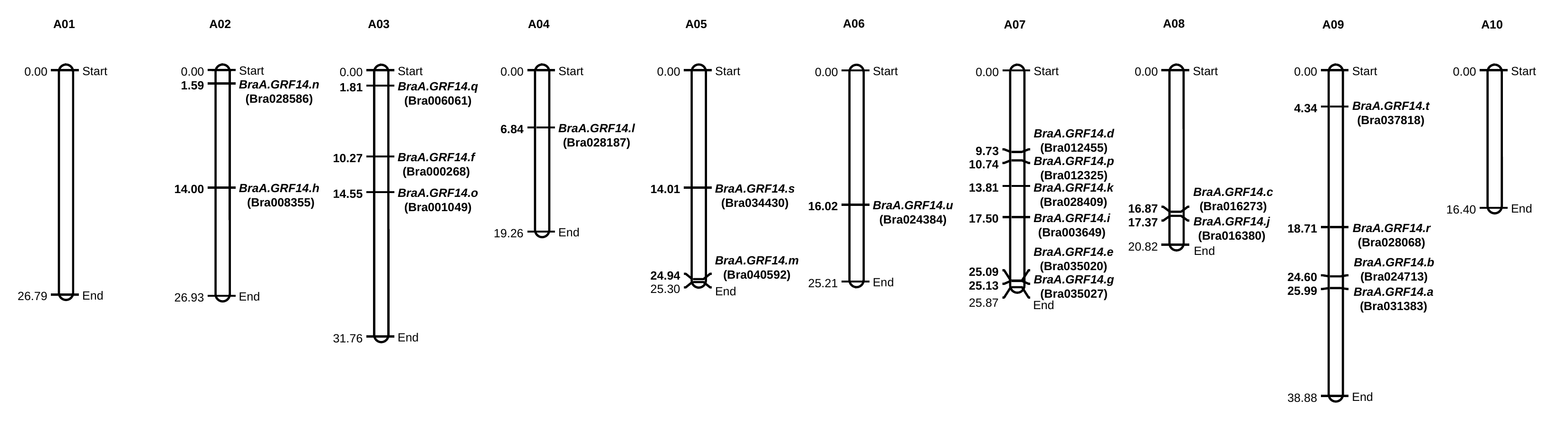

A08
Start
0.00
BraA.GRF14.c
(Bra016273)
16.87
BraA.GRF14.j
(Bra016380)
17.37
20.82
End
A06
Start
0.00
BraA.GRF14.u
(Bra024384)
16.02
End
25.21
A04
Start
0.00
BraA.GRF14.l
(Bra028187)
6.84
End
19.26
A05
Start
0.00
BraA.GRF14.s
(Bra034430)
14.01
BraA.GRF14.m
(Bra040592)
24.94
25.30
End
A01
Start
0.00
End
26.79
A02
Start
0.00
BraA.GRF14.n
(Bra028586)
1.59
BraA.GRF14.h
(Bra008355)
14.00
End
26.93
A03
Start
0.00
BraA.GRF14.q
(Bra006061)
1.81
BraA.GRF14.f
(Bra000268)
10.27
BraA.GRF14.o
(Bra001049)
14.55
End
31.76
A09
Start
0.00
BraA.GRF14.t
(Bra037818)
4.34
BraA.GRF14.r
(Bra028068)
18.71
BraA.GRF14.b
(Bra024713)
24.60
25.99
BraA.GRF14.a
(Bra031383)
End
38.88
A10
Start
0.00
End
16.40
A07
Start
0.00
BraA.GRF14.d
(Bra012455)
9.73
BraA.GRF14.p
(Bra012325)
10.74
13.81
BraA.GRF14.k
(Bra028409)
BraA.GRF14.i
(Bra003649)
17.50
BraA.GRF14.e
(Bra035020)
25.09
BraA.GRF14.g
(Bra035027)
25.13
25.87
End
