# Supplementary material for: Class-Specific Evolution and Transcriptional Differentiation of 14-3-3 Family Members in Mesohexaploid Brassica rapa
Source: Front Plant Sci. 2016 Jan 26;7:12. doi: 10.3389/fpls.2016.00012 (PMC4726770; doi:10.3389/fpls.2016.00012)
Supplement: Supplementary file 9 [file Presentation2.PPT]

## Slide 1
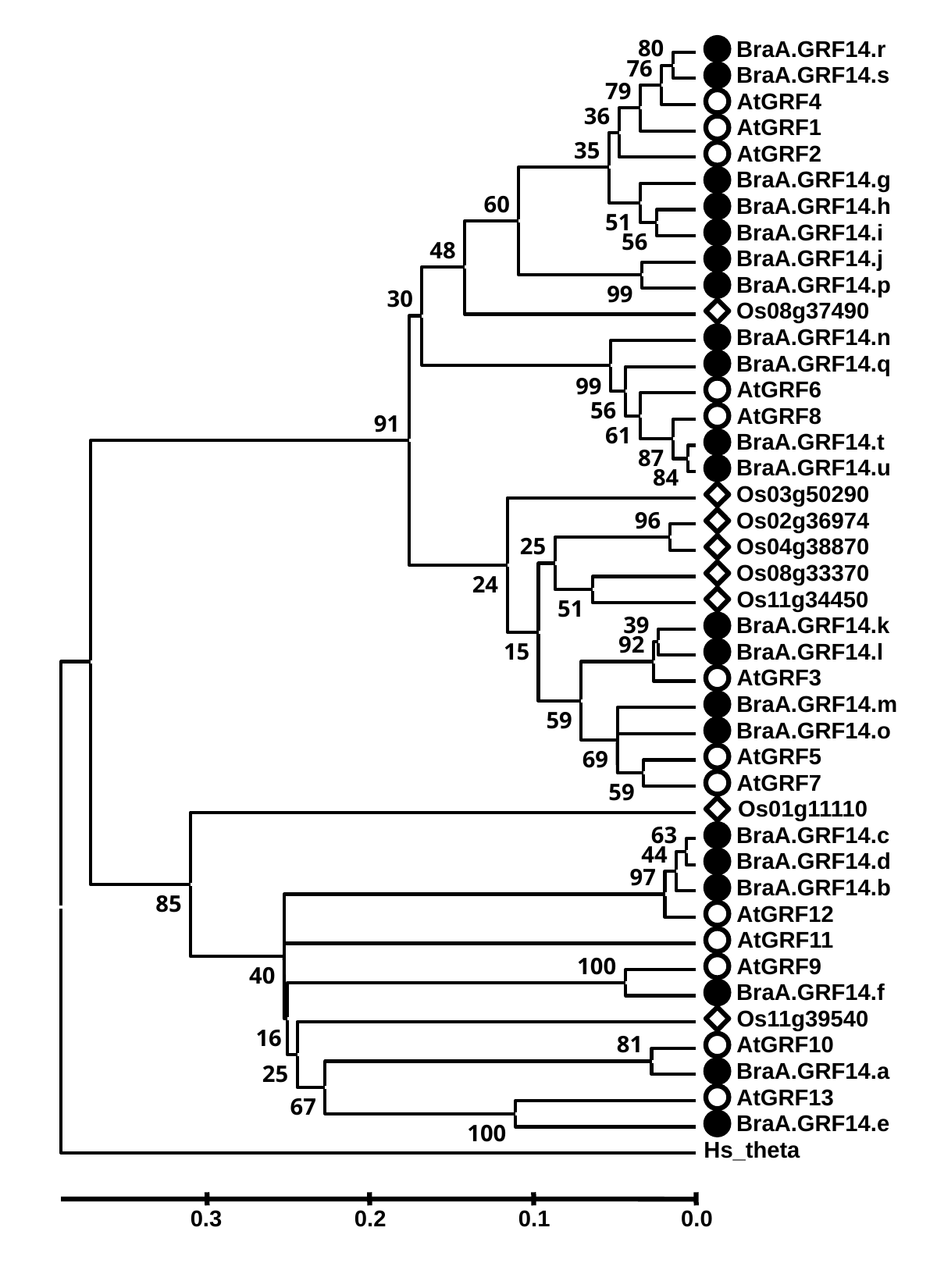

80
 BraA.GRF14.r
76
 BraA.GRF14.s
79
 AtGRF4
36
 AtGRF1
35
 AtGRF2
 BraA.GRF14.g
60
 BraA.GRF14.h
51
 BraA.GRF14.i
56
48
 BraA.GRF14.j
 BraA.GRF14.p
99
30
 Os08g37490
 BraA.GRF14.n
 BraA.GRF14.q
99
 AtGRF6
56
 AtGRF8
61
 BraA.GRF14.t
87
 BraA.GRF14.u
84
 Os03g50290
96
 Os02g36974
25
 Os04g38870
 Os08g33370
24
 Os11g34450
51
39
 BraA.GRF14.k
92
15
 BraA.GRF14.l
 AtGRF3
 BraA.GRF14.m
59
 BraA.GRF14.o
 AtGRF5
69
 AtGRF7
59
 Os01g11110
63
 BraA.GRF14.c
44
 BraA.GRF14.d
97
 BraA.GRF14.b
 AtGRF12
 AtGRF11
100
 AtGRF9
 BraA.GRF14.f
 Os11g39540
 AtGRF10
 BraA.GRF14.a
 AtGRF13
 BraA.GRF14.e
100
 Hs_theta
91
85
40
16
81
25
67
0.3
0.2
0.1
0.0
