# Supplementary material for: Class-Specific Evolution and Transcriptional Differentiation of 14-3-3 Family Members in Mesohexaploid Brassica rapa
Source: Front Plant Sci. 2016 Jan 26;7:12. doi: 10.3389/fpls.2016.00012 (PMC4726770; doi:10.3389/fpls.2016.00012)
Supplement: Supplementary file 10 [file Presentation3.PPT]

## Slide 1
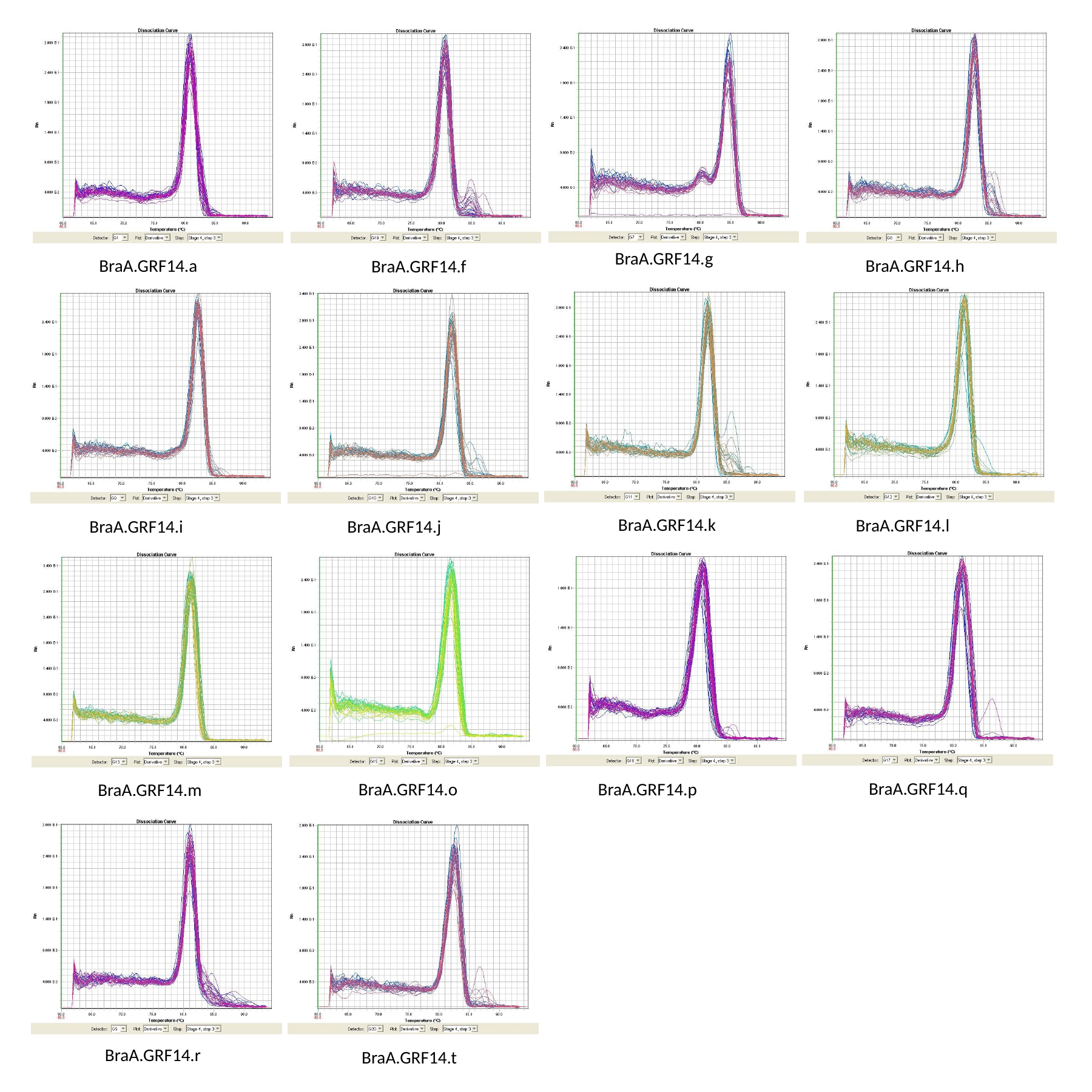

BraA.GRF14.g
BraA.GRF14.a
BraA.GRF14.h
BraA.GRF14.f
BraA.GRF14.k
BraA.GRF14.l
BraA.GRF14.i
BraA.GRF14.j
BraA.GRF14.q
BraA.GRF14.p
BraA.GRF14.o
BraA.GRF14.m
BraA.GRF14.r
BraA.GRF14.t
